# Time to viral load re-suppression and its predictors among adult patients on second-line anti-retro viral therapy in northeastern Ethiopia: multi-center prospective follow-up study

**DOI:** 10.3389/fmed.2025.1496144

**Published:** 2025-03-10

**Authors:** Abebe Yehualaw Melaku, Niguss Cherie, Tarikua Afework Birhanu, Muluken Amare Wudu

**Affiliations:** ^1^CDC Monitoring and Evaluation Officer at Dessie Comprehensive Specialized Hospital, Dessie, Ethiopia; ^2^Reproductive and Family Health Department, School of Public Health, College of Medicine and Health Sciences, Wollo University, Dessie, Ethiopia; ^3^Department of Pediatrics and Child Health Nursing, College of Medicine and Health Sciences, Wollo University, Dessie, Ethiopia

**Keywords:** viral load re-suppression, predictors, adult patient, second-line antiretroviral therapy, northeastern Ethiopia

## Abstract

**Background:**

Despite the increasing number of patients on second-line antiretroviral therapy in low-income countries such as Ethiopia, there is limited evidence regarding the time to viral re-suppression. Therefore, this study aimed to assess the time to viral load re-suppression and its predictors among adult patients on second-line antiretroviral therapy in northeastern Ethiopia.

**Method:**

A multi-center, institution-based prospective follow-up study was conducted over 48 months, from February 2022 to February 2024, involving 526 adults living with human immunodeficiency virus (HIV) who were receiving second-line antiretroviral therapy in northeastern Ethiopia. Data were collected through face-to-face interviews and chart reviews. A Weibull proportional hazards model was fitted to identify the predictors of viral re-suppression.

**Results:**

The median time to viral re-suppression was 9 months (IQR = 3–15 months). The rate of viral re-suppression was 44.3 per 1,000 person-months (95% CI: 40.4–49). Predictors of viral re-suppression included disclosure of Human Immunodeficiency Virus (HIV) status [AHR 2.24 (95% CI: 1.4–3.7)], classification in World Health Organization (WHO) clinical stages I and II [AHR 6.9 (95% CI: 4.4–9.6)], receipt of tuberculosis (TB) preventive treatment [AHR 3.7 (95% CI: 2.3–5.93)], absence of first-line drug substitution history [AHR 1.44 (95% CI: 1.2–1.8)], and good adherence to treatment [AHR 1.9 (95% CI: 1.4–2.54)].

**Conclusion and recommendations:**

In this study, the time to viral load re-suppression was longer than expected. Disclosure status, WHO clinical stage I or II, receiving tuberculosis preventive treatment, and the absence of first-line drug substitution history were predictors of viral load re-suppression. Health managers and antiretroviral therapy care providers must improve the timing and effectiveness of early disclosure, encourage the early use of tuberculosis prophylaxis, and maintain good adherence through various strategies.

## Introduction

In 2022, 39 million people worldwide were living with human immunodeficiency virus (HIV). Of these, 37.5 million were adults aged 15 and older (1, 2). That same year, we recorded 1.3 million new HIV infections, a global HIV incidence rate of 0.17 (a 32% reduction from 0.25 in 2015), and 630,000 HIV-related deaths (1, 2). In 2022, Africa achieved an impressive 53% reduction in HIV incidence. Despite this promising decline, Africa was projected to account for 50% of new HIV infections that year, making it the region with the highest HIV burden (1–3).

Global health sector strategies aim to achieve a 95% viral load suppression rate by 2025. However, in 2022, the actual implementation showed a rate of only 93%. Additionally, only 71% of HIV-positive people, regardless of age, had suppressed viral loads, indicating that further efforts are needed to reach the target (1–3). In 2023, in Ethiopia, a total of 605,238 people living with HIV were reported. Among these, 7,428 were new HIV infections, 513,990 (94%) people living with HIV knew their status and were currently on treatment, and 495,519 (96%) were virally suppressed, respectively (3).

For individuals resistant to first-line antiretroviral therapy (ART), second-line ART involves at least three active medications (TDF + 3TC + LPV/r), one of which should belong to a novel category to maximize therapeutic effectiveness and limit the risk of cross-resistance (4, 5). Despite the growing proportion of patients transitioning to second-line ART in Sub-Saharan Africa, treatment failure on second-line ART poses a barrier to maintaining viral suppression (6, 7).

Viral re-suppression is defined as the inability of the virus to multiply in the patient’s blood, with the viral load consistently maintained below 1,000 copies/mL or the virus being undetectable in the blood during a specific period of treatment for patients on second-line ART (4, 8). Consequently, an undetectable viral load increases the drive of patients to take drugs and follow-up care, and it is a key signal of treatment success (8). Time to viral load re-suppression is defined as the duration required to achieve viral load re-suppression following the initiation of second-line ART (8).

Recent research indicates that over 50% of second-line ART patients in developing countries fail to achieve or sustain persistent viral re-suppression (9). In Ethiopia, patients receiving second-line treatment make up 1.5% of the entire ART population (10). Approximately one-third of adults living with HIV in rural areas fail to achieve viral suppression, and viral load suppression in certain parts of the country remains low (11). Consequently, more effort is needed to improve the prompt and appropriate use of viral load values to inform clinical decisions for patients on ART (12).

Virological suppression differs based on the environment and patient characteristics. Various studies have shown that achieving and maintaining viral suppression depends on patient sociodemographic variables, viral load count at switch, and CD4 count at switch (9, 13–16). Furthermore, a history of tuberculosis (TB), TB preventive management, smoking habits, alcohol use, medication adherence, and baseline resistance to 3TC/TDF were predictors of viral load re-suppression among adults on second-line ART (17–19).

Global efforts aim to achieve three key objectives: 95% HIV diagnosis, 95% ART coverage, and 95% viral load suppression. Additionally, the goal is to reduce HIV incidence rates from 0.05 to 0.025, corresponding to 370,000 cases by 2025 and 335,000 cases by 2030 (20, 21). Ethiopia has also adopted and implemented this program, including launching a national guideline on HIV prevention, care, and treatment in 2022 (8).

Despite the significant increase in the number of patients on second-line ART in Ethiopia, there is a lack of evidence regarding the time to viral re-suppression and its predictors among adults on second-line ART. Only one study has focused on the time to viral re-suppression and its predictors in Ethiopia (13). In contrast to the previous study, the current study utilized a larger sample size, conducted a prospective follow-up, and used a parametric model.

As a result, assessing the time to viral re-suppression and its predictors among adults on second-line ART would provide valuable information to stakeholders about the current virological status of second-line ART users and assist in planning for future third-line ART needs. This survey could also be used to track progress toward achieving one-third of the UNAIDS 95–95-95 target by 2025. Therefore, this study aimed to determine the median time to viral load re-suppression and its predictors among adult patients on second-line anti-retroviral therapy in northeastern Ethiopia.

## Materials and methods

### Study setting, design, and period

A multi-center, prospective follow-up study was conducted at Dessie and Woldiya Comprehensive Specialized Hospitals in northeastern Ethiopia from 10 February 2022 to 22 February 2024. Northeastern Ethiopia has contributed to a high HIV burden, particularly in the Dessie area, where the prevalence was 8.5% and the incidence was 5.74 per 1,000 population, the highest in the Amhara region (22). Currently, a total of 566 adult patients are on second-line ART at Dessie and Woldiya Comprehensive Specialized Hospitals.

### Study population and eligibility criteria

All adults living with HIV who are on second-line ART and attending Dessie and Woldiya Comprehensive Specialized Hospitals were considered part of the study population. The inclusion criteria included adults living with HIV who were on second-line ART and had complete data. However, patients with incomplete data, including missing second viral load test results, were excluded from the study.

### Sample size determination and sampling procedures

We included all people living with HIV on second-line ART attending Dessie and Woldiya Comprehensive Specialized Hospitals from February 2022 to February 2024, as a final sample size. A total of 566 clients were on second-line ART during the study period in both hospitals. Out of the 566 participants, 526 were included in the study. Forty participants were excluded due to incomplete data, including missing second viral load test results and other essential variables.

### Study variables

The dependent variable was time to viral load re-suppression.

### Independent variables

Sociodemographic variables include age, sex, occupation, marital status, and residence.

Clinical and laboratory test-related factors include: disclosure status, BMI, functional status, WHO clinical stage, CD4 cell count (cells/mm^3^), and viral load.

ART and treatment-related factors include: medication adherence, TB treatment status at regimen switch, TB preventive status (had not taken/had taken), history of first-line drug substitution, and history of second-line drug substitution.

### Operational definitions

**Disclosure Status**: The extent to which an individual living with HIV has shared their HIV-positive status with others. This could be any individual, including at least one family member, partner, friend, or healthcare provider (8).

Viral load re-suppression was defined as a viral load count of less than 1,000 copies/mL or an undetectable viral load following a specified duration of treatment (4, 8, 23).

Time to viral load re-suppression was defined as the duration required to achieve viral load re-suppression following the initiation of second-line ART. For patients who did not achieve re-suppression, it was measured as the time elapsed from the start of second-line ART until their final recorded viral load measurement (in months).

The beginning time was defined as the date of initiation of second-line ART. The ending time was defined as the date of confirmed viral suppression for those who achieved it and the date of the last recorded measurement for patients who did not achieve viral suppression.

Censored cases included patients who died, were lost to follow-up, were transferred to another treatment site, developed any other outcome without achieving viral re-suppression, or had not achieved viral re-suppression by the end of the study period.

### Data collection methods and procedures

The data tool was adapted from the Federal Ministry of Health’s national guidelines for comprehensive HIV prevention care and management, chronic HIV follow-up forms, and HIV intake forms (8). The data were collected using face-to-face interviews and a structured checklist to review ART cohort registration records. Sociodemographic and behavioral factor data were collected through face-to-face interviews, while viral load re-suppression status data were obtained from viral load registration books. The registration records included chronic ART follow-up forms and routinely collected follow-up data from ART cohort registrations, ART intake forms, and viral load registration books. Two trained nurses and two medical doctors working in the ART clinic at each study hospital were hired as data collectors and supervisors, respectively.

### Data quality management

Data quality was ensured by developing a clear and straightforward data extraction checklist aligned with current chronic HIV care follow-up formats. Data collectors and supervisors received 3 days of training on the study’s purpose, data extraction methods from patient follow-up forms and registration books, and proper data recording techniques. Furthermore, supervisors monitored the data collection process to ensure its completeness.

### Data processing and analysis

The data were coded, entered, and cleaned using EpiData version 4.6, and then exported to STATA version 17 for analysis. Descriptive statistics, including means, medians, and frequency tables, were used to describe the characteristics of the study participants.

The proportional hazards assumption was assessed using a log–log plot and the log-rank test, which revealed both graphical and statistical differences in survival between variable categories. Kaplan–Meier curve analysis was used to illustrate the pattern of viral load re-suppression and to compare survival curves across different categories. Finally, the Schoenfeld residual test (global test) was used to evaluate the model’s goodness of fit.

For model comparison, the criteria set were: selecting the model with the highest log-likelihood, and the lowest Akaike Information Criterion (AIC) and Bayesian Information Criterion (BIC) values as the best fit. Consequently, the Weibull proportional hazards model was chosen over the others due to its higher log-likelihood (−721.6) and lower AIC (1465.2) and BIC (1512.1) values (Supplementary file 1).

The Weibull proportional hazard (PH) model was used to identify predictors of time to viral re-suppression. Variables with a *p*-value less than 0.20 in the bivariable analysis were considered candidates for multivariable Weibull PH regression. Ultimately, predictors of time to viral re-suppression were identified based on the adjusted hazard ratio (AHR) with a 95% confidence interval and statistical significance (*p* < 0.05). Multicollinearity was assessed using the variance inflation factor (VIF), which ranged from 2 to 4.2, indicating low multicollinearity. Additionally, the overall goodness of fit was evaluated using the Schoenfeld residual test (χ^2^ = 18.76, *p*-value = 0.73). The test result, being not statistically significant, suggested a good fit for the model.

## Results

### Sociodemographic characteristics of the study participants

Of the 526 participants, the majority were between 35 and 39 years old (140 participants, 26.6%). The mean age of the participants was 38.5 years (± 10 SD). Of the total participants, 294 (55.9%) were female, 342 (65%) were urban residents, 301 (57.2%) were married, 204 (38.8%) were unable to read or write, and 114 (21.7%) were housewives (Table 1).

**Table 1:** Sociodemographic characteristics of adult patients on second-line ART in northeastern Ethiopia, 2024 (*n* = 526).

Variables	Censored	Viral load re-suppressed
Age of client		
18–19 years old	9(9.2%)	27(6.3%)
20–24 years old	11(11.2%)	27(6.3%)
25–29 years old	10(10.2%)	18(4.2%)
30–34 years old	10(10.2%)	42(9.8%)
35–39 years old	22(22.4%)	118(27.6%)
40–44 years old	12(12.2%)	78(18.2%)
45–49 years old	9(9.2%)	42(9.8%)
≥ 50 years old	15(15.3%)	76(17.8%)
Sex		
Female	49(50%)	245(57.2%)
Male	49(50%)	183(42.8%)
Residence		
Urban	49(50%)	293(68.5%)
Rural	49(50%)	135(31.5%)
Marital status		
Married	52(53.1%)	249(58.2%)
Single	38(38.8%)	101(23.6%)
Divorced	5(5.1%)	52(12.1%)
Widowed	3(3.1%)	26(6.1%)
Educational status		
Unable to read and write	37(37.8%)	167(39%)
Primary education	39(39.8%)	163(38.1%)
Secondary education	17(17.3%)	72(16.8%)
Tertiary education	5(5.1%)	26(6.1%)
Occupational status		
Civil servant	1(1%)	10(2.3%)
Housewife	24(24.5%)	90(21%)
Merchant	3(3.1%)	55(12.9%)
Farmer	12(12.2%)	46(10.7%)
Daily labor	58(59.2%)	224(52.3%)
Private employed	0(0%)	3(0.7%)

### Behavioral-related factors

Of the total participants, 400 (76%) did not use condoms, 56 (10.1%) consumed alcohol, and 184 (35%) chewed khat (Table 2).

**Table 2:** Clinical, immunological, and treatment-related characteristics of adult patients living with HIV on second-line ART in northeastern Ethiopia, 2024 (*n* = 526).

Variable	Censored	Viral load re-suppressed
Condom utilization		
Yes	24(34.7%)	92(21.5%)
No	64(65.3%)	336(78.5%)
Alcohol consumption		
Yes	32(32.7%)	21(4.9%)
No	66(67.3%)	407(95.1%)
Khat chewing		
Yes	49(50%)	135(31.5%)
No	49(50%)	293(68.5%)
Disclosure status		
Disclosed	81(82.7%)	411(96%)
Not disclosed	17(17.3%)	17(4%)
BMI status		
<18.5 kg/m^2^	24(34.7%)	92(21.5%)
≥18.5 kg/m2	64(65.3%)	336(78.5%)
Functional status		
Workable	82(83.7%)	398(93%)
Ambulatory	9(9.2%)	26(6.1%)
Bedridden	7(7.1%)	4(0.9%)
WHO clinical stages		
I and II	68(69.4%)	421(98.4%)
III and IV	30(30.6%)	7(1.6%)
CD4 cell/mm^3^		
<450 cell/mm^3^	75(76.5%)	244(57%)
≥450 cell/mm^3^	23(23.5%)	184(43%)
Viral load count		
< 1,000 copy/ml	0(0%)	428(100%)
≥1,000 copy/ml	98(100%)	0(0%)
Medication adherence		
Good adherence ≥95%	77(78.6%)	382(89.3%)
Fair and Poor adherence <95%	21(21.4%)	46(10.7%)
TB treatment status at regimen switch		
On Anti-TB treatment	10(10.2%)	3(0.7%)
Not on Anti-TB treatment	88(89.8%)	425(99.3%)
TB preventive status		
Had not taken	32(32.7%)	21(4.9%)
Had taken	66(67.3%)	407(95.1%)
Had First-line drug substitution history		
Yes	40(40.8%)	129(30.1%)
No	58(59.2%)	299(69.9%)
Had Second-line drug substitution history		
Yes	55(56.1%)	121(28.3%)
No	43(43.95)	307(71.7%)

### Clinical and laboratory test characteristics of the study participants

The majority of participants disclosed their status (492 participants, 93.5%), had a BMI of ≥18.5 kg/m^2^ (400 participants, 76%), had a workable functional status (480 participants, 60.6%), were classified in WHO clinical stages I and II (489 participants, 93%), had a CD4 count of <450 cells/mm^3^ (319 participants, 60.6%), and had a viral load count of <1,000 copies/ml (428 participants, 81.4%; Table 2).

### Treatment-related characteristics of the study participants

Regarding treatment-related features, 459 (87.3%) of the patients had good medication adherence (≥95%), while 513 (97.5%) were not on anti-TB treatment. Additionally, 473 (89.9%) had taken TB preventive measures, 357 (67.9%) had no history of first-line drug substitution, and 350 (66.5%) had no history of second-line drug substitution (Table 2).

### Incidence rate and time to viral load re-suppression

**Figure 1:**
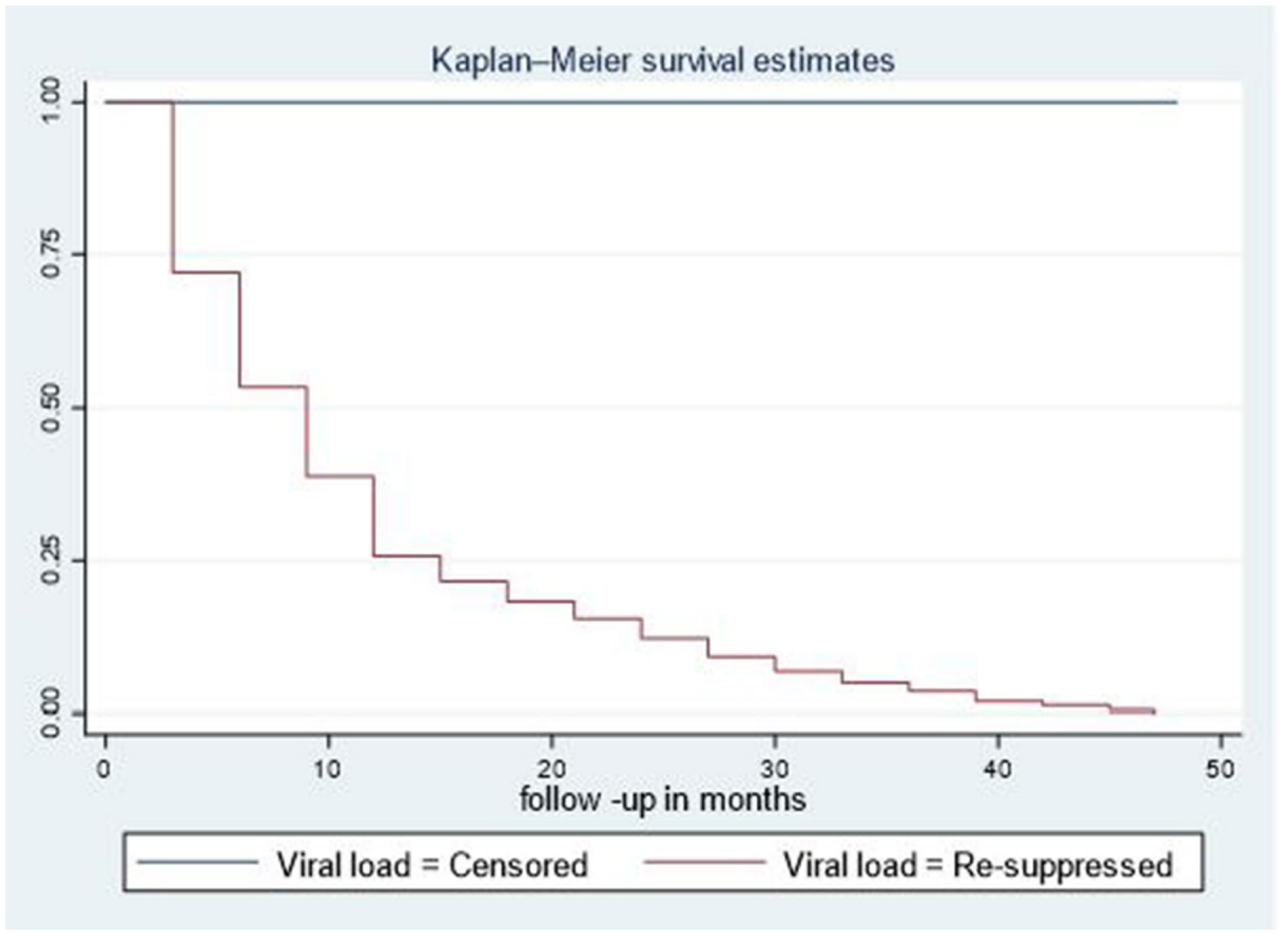
Median time to viral load re-suppression among adult patients living with HIV on second-line ART in northeastern Ethiopia, 2024 (*n* = 526).

Of the total participants, 428 (81.4%) had re-suppressed viral loads, 77 (14.6%) had unsuppressed viral loads, 12 (2.3%) were transferred out, 5 (0.95%) dropped out, and 4 (0.76%) died. The viral re-suppression rate was 44.3 per 1,000 (95% CI: 40.4–49) person-months of observation among second-line ART clients in northeastern Ethiopia, with a total of 9,646 person-months of observation. The median time to viral load re-suppression was 9 months (IQR = 3–15 months; Figure 1). The cumulative survival rate of second-line ART clients at the end of the 3rd, 9th, 15th, 30th, and 48th months was 77.2, 50.2, 36.12, 24.14, and 18.63%, respectively (Supplementary file 2).

### Time-to-viral re-suppression comparisons across different categorical variables

**Figure 2:**
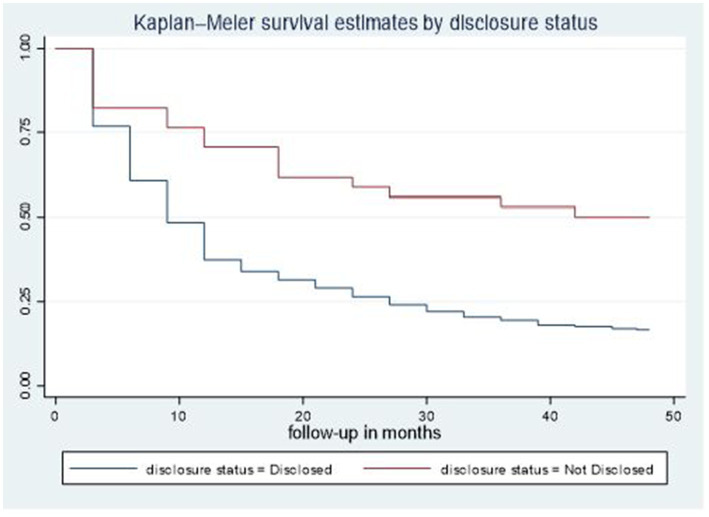
Time-to-viral re-suppression comparisons by disclosure status among adult patients living with HIV on second-line ART in northeastern Ethiopia, 2024 (*n* = 526).

The median time to viral load re-suppression hazard for second-line ART clients who had disclosed their status was 9 months (95% CI: 9–12), notably shorter than the median of 42 months observed for second-line ART clients not disclosed their status. Additionally, the disparity in survival rates was statistically significant (*p* = 0.001) based on the log-rank test (Figure 2).

**Figure 3:**
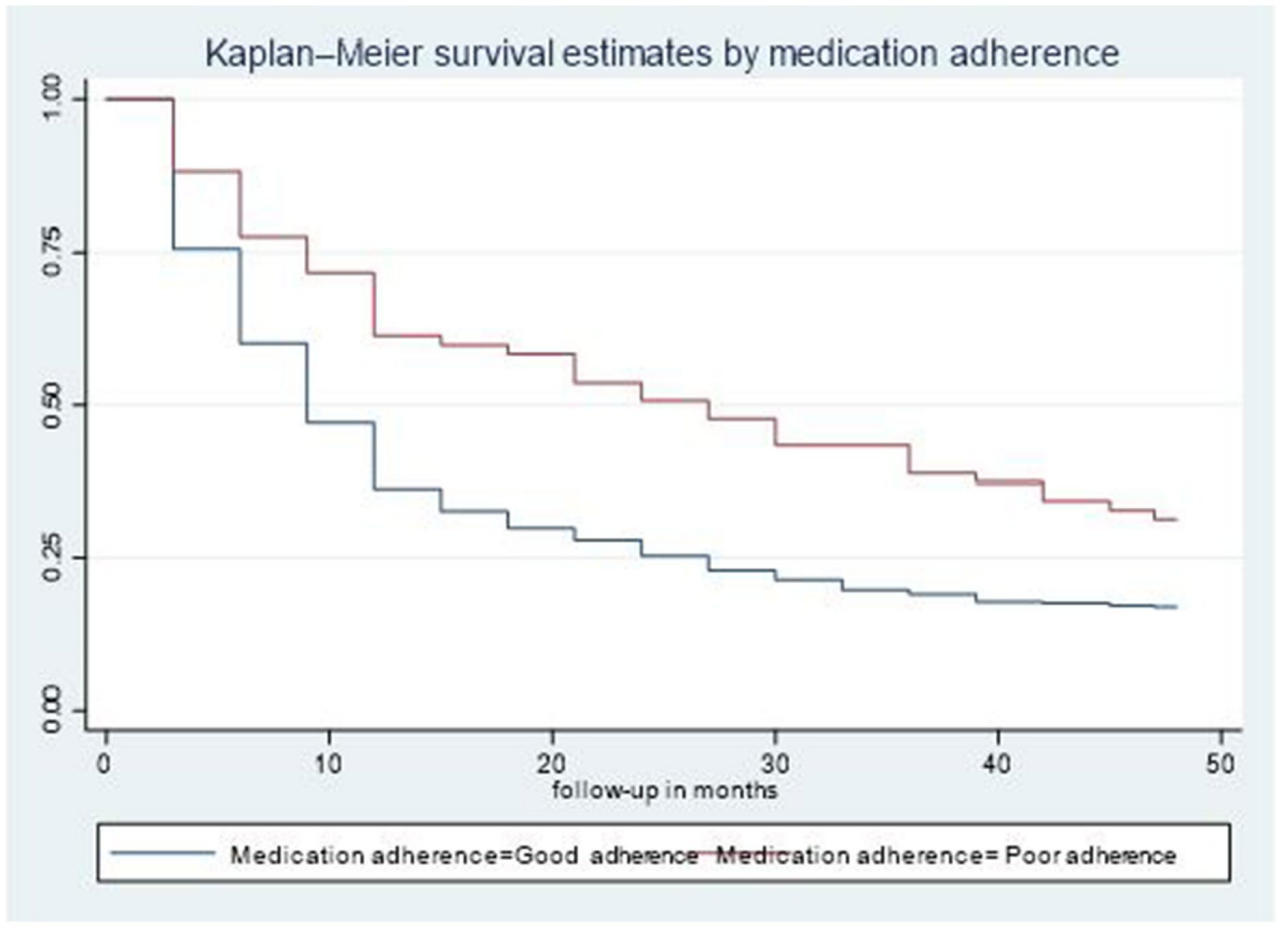
Time-to-viral re-suppression comparisons by medication adherence among adult patients living with HIV on second-line ART in northeastern Ethiopia, 2024 (*n* = 526).

The median time to viral load re-suppression hazard for second-line ART clients who had good medication adherence was 9 months (95% CI: 9–12), notably shorter than the median of 27 months observed for second-line ART clients who had poor medication adherence. Additionally, the disparity in survival rates was statistically significant (*p* = 0.003) based on the log-rank test (Figure 3).

### Predictors of time to viral load re-suppression

In the bivariable Weibull proportional hazard regression analysis, several factors were identified as predictors of time to viral load re-suppression among second-line ART clients. These factors included rural residence, disclosed status, workable and ambulatory functional status, WHO clinical stages I and II, TB preventive treatment, no first-line drug substitution history, and good adherence. After conducting the bivariable Weibull proportional hazard regression analysis, factors with *p*-values less than 0.25 were transformed into the multivariable Weibull proportional hazard regression.

In the multivariable analysis, rural residence, disclosed status, WHO clinical stages I and II, TB preventive treatment, no first-line drug substitution history, and good adherence were revealed as predictors of viral load re-suppression among second-line ART clients at Dessie Comprehensive Specialized Hospital.

During the follow-up period, second-line ART clients who had disclosed their status had a time to viral load re-suppression that was two times shorter (95% CI: 1.4–3.7) compared to their counterparts. Similarly, second-line ART clients who were classified in WHO clinical stages I and II re-suppressed their viral load approximately seven times as quickly (95% CI: 4.4–9.6) compared to those who were in WHO clinical stages III and IV. Moreover, second-line ART clients who had taken their TB preventive treatment had approximately four times shorter time to viral load re-suppression (95% CI: 2.3–5.93) compared to their counterparts. Similarly, second-line ART clients who had no first-line drug substitution history had a 1.44 times shorter time to viral load re-suppression (95% CI: 1.2–1.8) compared to their counterparts. Finally, second-line ART clients who had good adherence had a time to viral load re-suppression that was approximately two times shorter (95% CI: 1.4–2.54) compared to their counterparts (Table 3).

**Table 3:** Predictors of viral load suppression among adult patients living with HIV on second-line ART in northeastern Ethiopia, 2024 (*n* = 526).

Variables	Viral load re-suppression		CHR (95% CI)	AHR (95% CI)	*p*-value
	Censored	Viral load re-suppressed			
Residence					
Rural	49(50%)	135(31.5%)	0.68(0.56, 0.84)*	0.8(0.63, 0.95)**	0.016
Urban	49(50%)	293(68.5%)	reference	reference	reference
Disclosure status					
Disclosed	81(82.7%)	411(96%)	2.9(1.8, 4.7)*	2.24(1.4, 3.7)**	0.001
Not disclosed	17(17.3%)	17(4%)	reference	reference	reference
Functional status					
Workable	82(83.7%)	398(93%)	4.5(1.7, 9.2)*	2.04(0.76, 5.52)	0.16
Ambulatory	9(9.2%)	26(6.1%)	3.6(1.3, 9.3)*	2.24(0.78, 6.5)	0.134
Bedridden	7(7.1%)	4(0.9%)	reference	reference	reference
WHO clinical stages					
I and II	68(69.4%)	421(98.4%)	7.9(5.1, 9.81)*	6.9(4.4, 9.6)**	0.000
III and IV	30(30.6%)	7(1.6%)	reference	reference	reference
TB preventive status					
Had not taken	32(32.7%)	21(4.9%)	reference	reference	reference
Had taken	66(67.3%)	407(95.1%)	4.2(2.7, 6.5)*	3.7(2.3, 5.93)**	0.000
Had first-line drug substitution history					
Yes	40(40.8%)	129(30.1%)	reference	reference	reference
No	58(59.2%)	299(69.9%)	1.4(1.1, 1.7)*	1.44(1.2, 1.8)**	0.001
Medication adherence					
Good adherence	77(78.6%)	382(89.3%)	1.8(1.4, 2.52)*	1.9(1.4, 2.54)**	0.000
Fair and Poor adherence	21(21.4%)	46(10.7%)	reference	reference	reference

*Stands for statistical significance in bivariable analysis and ** for multivariable analysis.

## Discussion

This study aimed to assess time to viral load re-suppression and its predictors among adult patients living with HIV on second-line ART in northeastern Ethiopia. The findings of this study indicated that the median time to viral re-suppression after the initiation of second-line ART was 9 months (IQR = 3–15 months), which is longer than the expected 6 months for viral re-suppression, based on the UNAIDS guideline recommendations for low-resource settings and the Ethiopian Ministry of Health National HIV Care and Management Guidelines, which expect viral load re-suppression to occur within 3 to 6 months of starting second-line ART. This figure is similar to findings from studies in Kenya, Mozambique, and Ethiopia (13, 24, 25). A possible explanation could be the lack of routine viral load testing in accordance with guidelines, as well as the patients’ adherence levels and other clinical characteristics. This indicates that routine viral load testing is crucial for determining the exact time to viral re-suppression (4, 23) and for achieving the third component of the UNAIDS 95–95-95 target, which aims for 95% of ART clients to achieve viral load re-suppression (4). As a result, it would be preferable to conduct a prospective cohort study on second-line ART patients to assess the actual time to viral re-suppression after switching to second-line therapy.

In the current study, second-line ART patients who had disclosed their status experienced a shorter time to viral re-suppression compared to those who had not disclosed. This is in line with the result from Mozambique (25). This can be explained by the fact that early disclosure of their status might help patients adhere more strictly to their medication and adopt a positive lifestyle, leading to quicker viral load re-suppression (4, 8, 23). This implies that ART care providers need to encourage disclosure from the time of linkage to ART. Sharing one’s HIV status with at least one family member may help improve adherence. Consequently, viral load re-suppression was achieved within 3 months. This is crucial for both the individual’s health and minimizing the further spread of HIV (4, 23).

Similarly, second-line ART patients in WHO clinical stages I and II achieved viral load re-suppression approximately seven times faster than those in stages III and IV. These findings are consistent with studies from Eastern Europe, South Africa, and India (14–16). This can be explained by the fact that as a patient’s WHO clinical stage worsens, the likelihood of acquiring immune-related infections and other comorbidities increases. This, in turn, significantly raises the risk of treatment failure and slows the progress of viral load suppression (4, 8). This implies that care providers should focus on strategies that shorten the time to viral re-suppression and delay the progression to clinical stages III and IV.

Second-line ART patients who received TB preventive treatment experienced a much shorter time to viral load re-suppression compared to those who did not. This finding is aligned with results from southern Ethiopian studies (26, 27). This may be because early TB prevention can significantly reduce the risk of reactivating dangerous bacterial infections that damage the immune system. Early TB prophylaxis minimizes comorbidities and significantly lowers viral load (8). This suggests that all HIV-positive patients who do not have active TB, regardless of their CD4 count, should be given 6 months of isoniazid as TB prophylaxis to achieve the target of 95% viral load suppression.

Second-line ART clients with good adherence achieved viral load re-suppression approximately twice as quickly as their counterparts. This finding is consistent with data from Eastern Europe, South Africa, India, and Ethiopia (13–16). This could be explained by good ART adherence, where patients take nearly all of their prescribed medications. This adherence reduces viral replication, lowers viral loads, and increases CD4 counts (28–30). This implies that health managers and ART care providers need to sustain good adherence through various methods, including peer support and counseling. Additionally, mobile phone interventions, such as medication trackers, SMS reminders, and educational messages, can enhance adherence to ART.

Finally, second-line ART clients who had no first-line drug substitution history achieved viral load re-suppression approximately twice as quickly as their counterparts. The finding is similar to the results from studies in Ethiopia (18, 19). The possible explanation might be that having fewer complications and comorbidities, along with a good support system, significantly decreases the probability of first-line drug substitution (26–28). It highlights the need for improved adherence support, close monitoring for resistance, and tailored treatment approaches to enhance the effectiveness of second-line ART.

### Strengths and limitations of this study

The study was conducted across multiple centers with a sufficiently large sample size, and patients were followed for up to 48 months, which enhances the generalizability of the findings. However, qualitative data were not included to explore the predictors of viral load re-suppression, which are not addressed in the quantitative data.

## Conclusion and recommendations

In this study, the time to viral load re-suppression was longer than expected. Disclosure of patients’ status, classification in WHO clinical stages I and II, receipt of TB prophylaxis, no history of first-line drug substitution, and good adherence were predictors of viral load re-suppression among adult patients on second-line ART. Health managers and ART care providers must improve the timing and effectiveness of early disclosure, encourage the early use of TB prophylaxis, and maintain good adherence through various strategies. Moreover, other researchers need to conduct a mixed-methods study to explore additional predictors of viral load re-suppression that are not addressed in the quantitative data.

## Data Availability

The original contributions presented in the study are included in the article/Supplementary material, further inquiries can be directed to the corresponding author.
