# Supplementary material for: Time to viral load re-suppression and its predictors among adult patients on second-line anti-retro viral therapy in northeastern Ethiopia: multi-center prospective follow-up study
Source: Front Med (Lausanne). 2025 Mar 10;12:1496144. doi: 10.3389/fmed.2025.1496144 (PMC11930823; doi:10.3389/fmed.2025.1496144)
Supplement: Supplementary file 1 [file Table_1.docx]

**Table: Model diagnosis and summary**

| **Models** | **N** | **Log likelihood** | **AIC** | **BIC** |
| --- | --- | --- | --- | --- |
| Cox | 526 | -2394.5 | 4807.01 | 4845.4 |
| Exponential | 526 | -757.9 | 1535.8 | 1578.43 |
| Gomperz | 526 | -747.12 | 1516.23 | 1563.2 |
| **Weibull** | **526** | **-721.6** | **1465.2** | **1512.1** |
| lognormal | 526 | -757.8 | 1537.6 | 1584.5 |
