# Supplementary material for: Time to viral load re-suppression and its predictors among adult patients on second-line anti-retro viral therapy in northeastern Ethiopia: multi-center prospective follow-up study
Source: Front Med (Lausanne). 2025 Mar 10;12:1496144. doi: 10.3389/fmed.2025.1496144 (PMC11930823; doi:10.3389/fmed.2025.1496144)
Supplement: Supplementary file 2 [file Table_2.docx]

**Supplementary file 2: life table cumulative survival probability among adult patients living with HIV on second line ART in northeastern Ethiopia, 2024 (n=526)**

| **Interval** | | **Beg. total** | **death** | **lost** | **Survival** | **Std. Error** | **[95% CI]** | |
| --- | --- | --- | --- | --- | --- | --- | --- | --- |
| 3 | 4 | 526 | 120 | 0 | 0.7719 | 0.0183 | 0.7336 | 0.8054 |
| 6 | 7 | 406 | 79 | 0 | 0.6217 | 0.0211 | 0.5787 | 0.6616 |
| 9 | 10 | 327 | 63 | 0 | 0.5019 | 0.0218 | 0.4584 | 0.5438 |
| 12 | 13 | 264 | 56 | 0 | 0.3954 | 0.0213 | 0.3536 | 0.4370 |
| 15 | 16 | 208 | 18 | 0 | 0.3612 | 0.0209 | 0.3203 | 0.4022 |
| 18 | 19 | 190 | 14 | 0 | 0.3346 | 0.0206 | 0.2946 | 0.3750 |
| 21 | 22 | 176 | 12 | 0 | 0.3118 | 0.0202 | 0.2726 | 0.3516 |
| 24 | 25 | 164 | 14 | 0 | 0.2852 | 0.0197 | 0.2472 | 0.3242 |
| 27 | 28 | 150 | 13 | 0 | 0.2605 | 0.0191 | 0.2237 | 0.2986 |
| 30 | 31 | 137 | 10 | 0 | 0.2414 | 0.0187 | 0.2058 | 0.2788 |
| 33 | 34 | 127 | 8 | 0 | 0.2262 | 0.0182 | 0.1915 | 0.2628 |
| 36 | 37 | 119 | 6 | 0 | 0.2148 | 0.0179 | 0.1808 | 0.2508 |
| 39 | 40 | 113 | 7 | 0 | 0.2015 | 0.0175 | 0.1684 | 0.2368 |
| 42 | 43 | 106 | 3 | 0 | 0.1958 | 0.0173 | 0.1631 | 0.2308 |
| 45 | 46 | 103 | 3 | 0 | 0.1901 | 0.0171 | 0.1578 | 0.2247 |
| 47 | 48 | 100 | 2 | 0 | 0.1863 | 0.0170 | 0.1543 | 0.2207 |
| 48 | 49 | 98 | 0 | 98 | 0.1863 | 0.0170 | 0.1543 | 0.2207 |
